# Fat embolism syndrome: A severe case diagnosed early using dual‐energy CT and saved with VA‐ECMO and inhaled nitric oxide

**DOI:** 10.1002/ccr3.8681

**Published:** 2024-03-29

**Authors:** Tsuyoshi Ota, Takahiro Sawada, Hiroyuki Shimoura, Yuya Terao, Tatsuro Ito, Katsunori Okajima, Makoto Kadotani, Yoshio Onishi

**Affiliations:** ^1^ Department of Cardiology Kakogawa Central City Hospital Kakogawa Hyogo Japan

**Keywords:** dual‐energy CT, fat embolism syndrome, inhaled nitric oxide, VA‐ECMO

## Abstract

Diagnosing FES is difficult and time‐consuming, and identify FES as an etiology of right ventricular volume overload for early diagnosis. Because FES is a reversible condition, even severe cases can bse treated if the patient survives the acute phase.

## Graphical abstract

Perfusion defects in dual‐energy CT are consistent with pulmonary infiltrates.

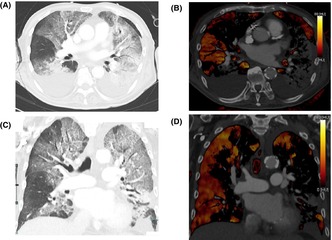

## 1 INTRODUCTION

Fat embolism syndrome (FES) is the release of bone marrow components into blood vessels, which can occur after long bones or pelvis fracture and can cause severe inflammatory conditions.^1^ Although several diagnostic criteria exist for FES, they require a wide range of clinical findings and the exclusion of other diseases, which is time‐consuming. Therefore, the early diagnosis of FES is challenging.

In this study, we aimed to discover a useful modality for early diagnosis of FES. From the perspective of pathophysiology of FES, we considered that dual‐energy computed tomography (DECT) may be useful for it. DECT can visualize the local iodinated contrast distribution in the lungs.^2^ It generally indicates pulmonary perfusion by analyzing two different types of x‐ray energy irradiation data.^2^ Therefore, DECT is commonly used for the diagnosis of pulmonary thrombo‐embolism (PTE).

In this case, we diagnosed a severe FES early by DECT and successfully treated with veno‐arterial extracorporeal membrane oxygenation (VA‐ECMO) and inhaled nitric oxide (iNO) therapy.

## 2 CASE HISTORY

An 86‐year‐old man was in a state of cardiac arrest 6 hours after a fall at home, and presented to our emergency department. The patient had a history of hypertension, type 2 diabetes mellitus, and pacemaker implantation for complete atrioventricular block that occurred 2 years ago.

Upon arrival at the emergency department, the patient  presented a Glasgow Coma Scale of 3/15 (E‐1,V‐1,M‐1), blood pressure of 110/70 mmHg, heart rate of 100 beats/min, oxygen saturation of 50% (under oxygen 15 L/min administration with bag‐valve‐mask ventilation) with agonal respiration, and body temperature of 35.4°C. The extremities were warm without edema, and both pupillary light reflexes and tendon reflexes were normal, with no seizures.

**FIGURE 1:**
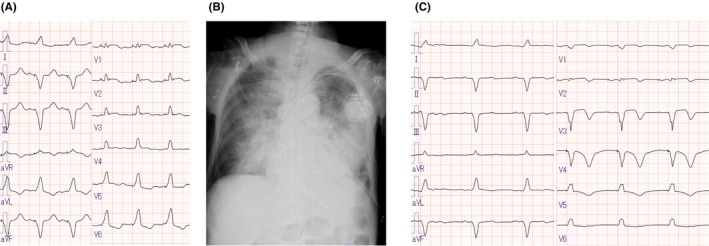
Electrocardiography (ECG) and chest radiography. (A) ECG at the presentation (Day 1). (B) Chest radiography at the presentation (Day 1) shows pulmonary infiltrates in both lungs. (C) ECG on Day 2 shows T wave inversion in the precordial leads between Days 1 and 2.

**FIGURE 2:**
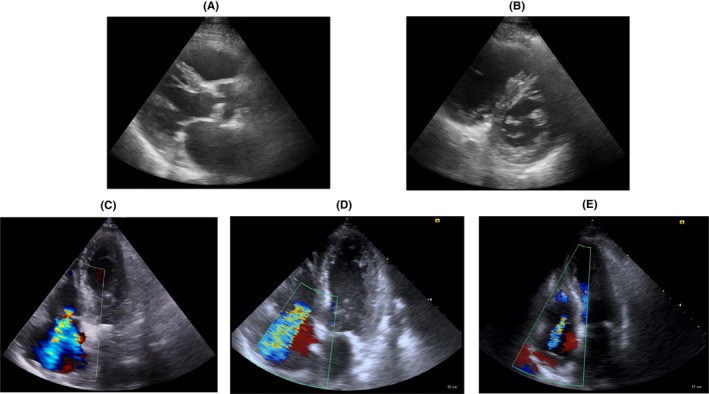
Transthoracic echocardiography. (A) Parasternal long‐axis view at the presentation (Day 1) (B) Apical 4‐chamber view at the presentation (Day 1) (A) and (B) show a significantly dilated right ventricle, suggesting right ventricular volume overload. (C) Tricuspid regurgitation (TR) jet at the presentation (Day 1). (D) TR jet 5 h after the presentation (Day 1) shows exacerbation. (E) TR jet on Day 5 shows marked improvement.

Additionally, 12‐lead electrocardiography (ECG) revealed sinus rhythm and atrial‐sensing ventricular‐pacing without ST‐segment changes (Figure 1A). Table 1 presents blood data. Arterial blood gas analysis  displayed marked hypoxemia, metabolic acidosis, and high lactate levels. Chest radiography indicated segmental infiltrative shadows and pleural effusion in both lungs. (Figure 1B). The patient was intubated shortly after presentation due to severe respiratory failure. A computed tomography (CT) image revealed a left femoral trochanteric fracture but showed no lesion in the brain that could cause a consciousness disorder. Transthoracic echocardiography (TTE) significantly dilated right atrium, right ventricle, and inferior vena cava (Figure 2A,B). Tricuspid regurgitation was severe, with 58 mmHg of the pressure gradient (Figure 2C). Left ventricular ejection fraction (LVEF) reduced to 40%, asynergy was observed in LV anterior and apical walls, and the LV morphology showed D‐shape due to a significant right ventricular volume overload (RVVO).

**TABLE 1:** Blood tests and arterial blood gas analysis at the presentation (Day 1).

Blood tests		Normal values
Hemoglobin, g/dL	8.9^a^	11.6–14.8
White blood cell count, ×10^3^/L	8.95^b^	3.3–8.6
Platelet count, ×10^3^/L	233	158–348
Sodium, mmol/L	143	138–145
Potassium, mmol/L	3.9	3.6–4.8
Aspartate aminotransferase, U/L	48^b^	13–30
Alanine aminotransferase, U/L	37	7–23
Lactate dehydrogenase, U/L	315^b^	124–222
C‐reactive protein, mg/dL	23.69^b^	<0.14
Total protein, g/dL	6.2^a^	6.6–8.1
Albumin, g/dL	2.8^a^	4.1–5.1
Blood urea nitrogen, mg/dL	21.1^b^	8–20
Creatinine, mg/dL	1.00^b^	0.46–0.79
Creatinine kinase, U/L	83	59–248
Creatinine kinase‐MB, g/L	8	<25
Brain natriuretic peptide, pg/mL	419.4^b^	<18.4
Troponin, pg/mL	142.01	<47.34
Thyroid stimulating hormone, IU/mL	1.73	0.61–4.23
Free thyroxine, ng/dL	1.43	0.77–1.59
Glucose, mg/dL	193^b^	73–109
Hemoglobin A1c, %	7.2^b^	4.9–6.0
Prothrombin, %	49.4^a^	70–130
Activated partial thromboplastin time, s	29.4	26–38
D‐dimer, g/mL	14.6^b^	<1.0

Arterial blood gas analysis (under oxygen 15 l/min)		Normal values
pH	7.210^a^	7.35–7.45
PaCO_2_, mmHg	39.9	35–48
PaO_2_, mmHg	17.6^a^	83–108
SaO_2_, %	Unmeasurable	95–99
Lactate, mmol/L	8.22^b^	0.5–1.6
HCO_3_^−^, mmol/L	15.6^a^	22.5–26.9

^a^ The value is less than normal.

^b^ The value is greater than normal.

## 3 DIFFERENTIAL DIAGNOSIS, INVESTIGATIONS, AND TREATMENT

**FIGURE 3:**
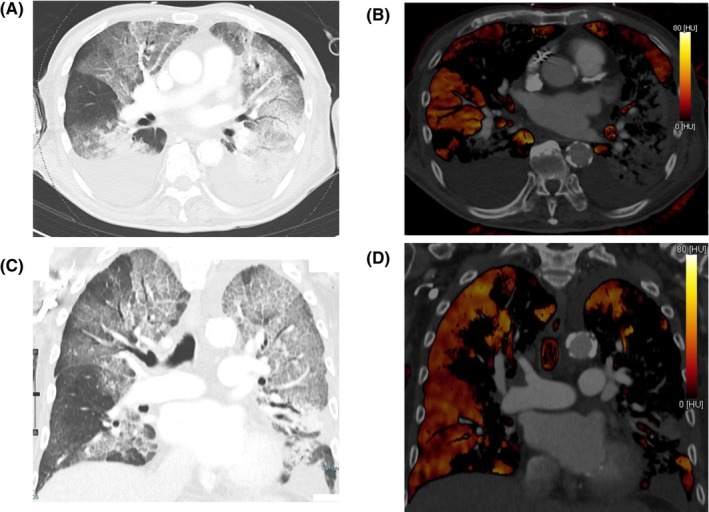
Plain CT and dual‐energy CT image at the presentation (Day 1). (A) Plain CT image of the transverse plane. (B) Dual‐energy CT image of the transverse plane. (C) Plain CT image of the coronal plane. (D) Dual‐energy CT image of the coronal plane. (A–D) show perfusion defects consistent with pulmonary infiltrates in both lungs.

Because of RVVO, CT pulmonary angiography (CTPA) was performed on the suspicion of PTE. No contrast defects were observed in the pulmonary arteries. Conversely, DECT images showed perfusion defects in the lungs consistent with pulmonary infiltrates (Figure 3). Since the patient had a femoral trochanteric fracture and the findings were mostly consistent with the pathophysiology of FES, FES was strongly suspected. The patient was promptly transferred to the intensive care unit, where dobutamine and norepinephrine were administered. However, the deterioration of oxygenation and reduction in blood pressure gradually progressed. 5 hours after arrival at the hospital, TTE revealed a worsening of RVVO and a reduction in LVEF to 15% (Figure 2D). Since FES was a reversible condition and the patient had a good chance of survival if he could survive the acute phase, VA‐ECMO was initiated in spite that the patient was of very old age.

## 4 OUTCOME AND FOLLOW‐UP

RVVO markedly improved after VA‐ECMO initiation. However, LVEF deterioration did not improve. Considering the absence of significant stenosis in coronary angiography and the subsequent clinical course, the possibility of Takotsubo cardiomyopathy (TC) as a complication was considered.

On the next day of admission (Day 2), because pulmonary vascular resistance (PVR) remained high after VA‐ECMO insertion, iNO was initiated and continued at a dose of 20 ppm to dilate the pulmonary arteries. Subsequently, the pulmonary artery, right ventricle, and central venous pressure decreased substantially. ECG showed T‐wave inversions in the precordial leads, similar to the natural course of TC (Figure 1C).

On Day 3, TTE showed improvement in LVEF to 30% and regional wall motion abnormalities of LV in the apex and mid‐ventricular segments consistent with TC. Since hemodynamic improvement was achieved, VA‐ECMO was removed, and an intra‐aortic balloon pump (IABP) was inserted.

On Day 4, LVEF improved to 45%, and chest radiography revealed that the pulmonary infiltrates had almost disappeared. After the removal of IABP, the patient was extubated and iNO was terminated.  Instead, oxygenation with a high‐flow nasal cannula (HFNC) was initiated.

On Day 5, the level of consciousness fully recovered, and oral food intake was initiated. On Day 7, HFNC was terminated, whereas RVVO improved significantly, and LVEF improved to 50% (Figure 2E). On Day 59, the patient was transferred to the hospital for rehabilitation. Unfortunately, the patient passed away due to septic shock originating from urinary tract infection at the hospital.

## 5 DISCUSSION

FES occurs in 0.3%–1.3% of traumatic injuries that result in fractures or require hospitalisation.^1^ FES typically causes embolic phenomena affecting multiple organs. Respiratory symptoms, central nervous system symptoms, and petechial hemorrhagic lesions are the classic triads.^1^ The mechanism of FES involves fat entering the venous system and reaching the pulmonary arteries. Our case met almost all diagnostic criteria of FES, including Tsuruta's criteria,^3^ Gurd and Wilson's criteria,^4^ and Schonfeld's scoring system^5^; however, a definitive diagnosis was not made until several days had passed. Currently, no specific findings of FES that allow for early diagnosis exist. The findings on susceptibility‐weighted imaging, one of the imaging modalities for brain magnetic resonance imaging (MRI) are specific to FES.^5^ However, its sensitivity is not high, and imaging may not be feasible at all in severe cases. Therefore, specific FES findings that can be assessed in severe cases are desirable.

Piolanti et al. demonstrated that plain CT findings are not specific for diagnosing FES.^6^ Furthermore, since FES is formed from small fat particles mainly distributed in the periphery,^7^ abnormal findings on CTPA are very rare.^8^ While it may be challenging to visualize the fat particles directly on imaging modalities due to their small size, we considered it possible to capture the phenomenon of decreased perfusion associated with their occlusion using DECT. Actually, Chun et al. reported in an animal model study that DECT provided higher sensitivity and accuracy in the detection of FES, as well as earlier detection than CTPA.^2^ In this study, FES was defined as perfusion defects on DECT, and histopathological results were used as the reference standard for evaluation.

In our case, plain CT showed segmental pulmonary infiltrates, and CTPA showed no contrast defects in the pulmonary artery. However, DECT revealed perfusion defects consistent with pulmonary infiltration. This finding is consistent with the pathophysiology of FES, in which fat particles embolize the pulmonary artery and induces inflammation around it. Therefore, we believe that DECT is highly useful for early diagnosis of FES.

A fat embolus could cause mechanical blockage in the pulmonary blood vessels and stimulate peripheral vascular endothelial cells to release lipase, which increase the free fatty acids level, thus leading to vascular permeability changes, pulmonary artery pressure increases, and blood vessel pathological changes.^9^

The treatment of FES is supportive without any specific therapeutic measures.^10^ In severe cases where circulatory dynamics have collapsed, the powerful cardiopulmonary support provided by ECMO can stabilize the hemodynamic status. There are multiple reported cases of successful resuscitation of severe FES using VA‐ECMO.^10^ In our case, VA‐ECMO was initiated due to collapse of circulatory dynamics.

Although definitive evidence is lacking, in an effort to reduce elevated PVR potentially caused by physiological changes such as inflammation, apart from mechanical obstruction of the vessels, iNO was initiated in our case. iNO is a safe and effective pulmonary vasodilator for acute PTE in an in vivo porcine model.^11^ However, only a few case reports on iNO use in patients with FES are available.^12^, ^13^ In our case, there was a significant improvement in RVVO after the initiation of iNO. Further research is required to confirm these findings.

It is considered that LV dysfunction in FES is caused by RVVO.^10^ In other words, RVVO cause displacement of the ventricular septum, resulting in a decrease of LV filling volume and subsequent LV dysfunction.^10^ Therefore, it is anticipated that by improving RVVO through the withdrawal of blood from right atrium and delivery to aorta using VA‐ECMO, LV function will recover to the level before the onset of FES. However, in our case, despite a significant improvement in RVVO following the initiation of VA‐ECMO, LVEF did not show any improvement. In our case, typical findings consistent with TC were observed on both TTE and ECG. The reversible decrease in LVEF further supports the typical nature of TC in this case. It is considered as the pathophysiology of TC, that excessive levels of catecholamines released by the sympathetic nervous system caused by a stressful condition could result in cardiotoxicity by intracellular calcium overload and microvascular dysfunction.^14^ In our case, it is considered that TC occured due to the severe physical stress of FES.

Conclusively, an elderly patient with severe FES could be successfully treated through early intensive care with VA‐ECMO and iNO based on the diagnosis of FES using DECT. DECT is considered highly helpful for the early diagnosis of FES.

## AUTHOR CONTRIBUTIONS

**Tsuyoshi Ota:** Writing – original draft. **Takahiro Sawada:** Writing – review and editing. **Hiroyuki Shimoura:** Writing – review and editing. **Yuya Terao:** Writing – review and editing. **Tatsuro Ito:** Writing – review and editing. **Katsunori Okajima:** Writing – review and editing. **Makoto Kadotani:** Writing – review and editing. **Yoshio Onishi:** Writing – review and editing.

## FUNDING INFORMATION

No financial support was received for this case report.

## CONFLICT OF INTEREST STATEMENT

The authors have no conflicts of interest to declare.

## CONSENT

Written informed consent was obtained from the patient to publish this report in accordance with the journal's patient consent policy.

## Data Availability

All data regarding this case has been reported in the manuscript. Please contact the corresponding author if you are interested in any further information.
